# Randomised clinical trial evaluating the effect of different timing and number of fixed timed artificial inseminations, following a seven-day progesterone-based protocol, on pregnancy outcomes in UK dairy heifers

**DOI:** 10.1136/vr.104369

**Published:** 2017-11-30

**Authors:** John Paul Walsh, Amy Coates, Fabio Lima, Rob Smith, Georgios Oikonomou

**Affiliations:** 1 Department of Livestock Health and Welfare, Institute of Veterinary Science, University of Liverpool, Leahurst, Neston, UK; 2 Delaware Veterinary Group, Fulford House, Castle Cary, Somerset, UK; 3 Department of Veterinary Clinical Medicine, University of Illinois Urbana, Champaign, IL, USA; 4 Department of Epidemiology and Population Health, Institute of Infection and Global Health, University of Liverpool, Leahurst, Neston, UK

**Keywords:** Dairy Heifer, Fixed Time Artificial Insemination, CIDR

## Abstract

The objective of this study was to determine the effect on pregnancy outcome of either inseminating heifers twice (at 48 and 72 hours after withdrawal of a controlled internal drug release insert (CIDR) containing progesterone) or once (56 hours after CIDR withdrawal) following a seven-day CIDR synchronisation protocol. Dairy heifers (n=267) from five farms, with an age range of 388–736 days, were randomly assigned to one of two treatment groups (group A heifers were inseminated twice; group B heifers were inseminated once). Both groups received a CIDR on day (D) 0 and an intramuscular injection of d-cloprostenol on D6; the CIDR was withdrawn on D7. Measurements of withers height, body condition score and hearth girth (used to estimate weight) were taken on D0. The diameter of the largest follicles and corpora lutea was recorded on both D0 and D6. Data were analysed with the use of multivariable logistic regression modelling. Treatment group and farm were not statistically significantly associated with pregnancy per treatment (P/T). Age and dominant follicle size on D6 were significantly associated with P/T. Heifers with the largest dominant follicle sizes (16–22 mm) were 5.54 times less likely to be pregnant than those heifers with the smallest dominant follicles (8–10 mm) on D6. It was shown that the cost associated with inseminating heifers twice after a seven-day CIDR synchronisation protocol is not justified.

## Introduction

The use of oestrous synchronisation and fixed time artificial insemination (FTAI) in dairy heifers can reduce the need for oestrus detection (OD). FTAI programmes have been shown to be economically viable when compared with inseminating to observed oestrus when heat detection rates were below 70 per cent.^1^ Silva and others^2^ showed that inseminating heifers for the first time using a progesterone-based synchronisation regimen resulted in lower cost per pregnancy and more pregnant animals after an 84-day breeding period compared with inseminating after OD alone; in this study the mean OD rate was 82 per cent.

Heifers that are non-cycling at the commencement of synchrony programmes are less likely to exhibit oestrus^3^ and have lower conception and pregnancy rates when compared with cycling heifers.^4^ In non-cycling beef heifers, inclusion of a progestogen (P4) in the synchrony programme (seven-day progesterone, prostaglandin F2α (PGF2α) administered on day (D) 6 post-P4 insertion) significantly increased submission and pregnancy rates compared with a single dose of PGF2α, or untreated controls, during a three-day breeding period where OD was carried out twice daily.^3^ However, the same study found that P4 treatment in dairy heifers did not result in increased submission and pregnancy rates compared with those achieved by the group of heifers receiving a single dose of PGF2α.^3^

Recent research has focused on combining variations of the Ovsynch protocol with progesterone inserts. In an attempt to prevent premature ovulation in dairy heifers, Rivera and others^5^ incorporated a progesterone insert from D0 to D6 into an Ovsynch or Co-Synch (gonadotropin-releasing hormone (GnRH) before FTAI or GnRH concurrent with FTAI, respectively) protocol, which suppressed ovulation during the days of progesterone presence. Research has also focused on reducing the period of follicular dominance, ensuring complete luteolysis and optimising the period of pro-oestrus to improve pregnancies per artificial insemination (P/AI). A reduced period of dominance of the ovulatory follicle has been reported to improve embryo quality^6^ and to reduce the chance of oocytes undergoing premature resumption of meiosis.^7^ Bridges and others^8^ showed that an extended period of pro-oestrus increased pregnancy rate after FTAI, and increased both preovulatory oestradiol and postovulatory progesterone concentrations. Recent studies have consistently reported P/AI ranging from 50 to 60 per cent in dairy heifers subjected to the five-day timed artificial insemination (AI) programme.^9 10^ The most consistent P/AI of 62 per cent was achieved by Lima and others,^11^ who increased ovulation by administration of GnRH at the initiation of the protocol, and improved luteolysis at insemination by using two doses of PGF2α.

The percentage of heifers showing oestrus after the controlled internal drug release (CIDR) insert containing progesterone removal following a seven-day CIDR synchronisation protocol was 7 per cent at 24 hours, 47 per cent at 36 hours and 20 per cent at 48 hours,^4^ and 25 per cent at 25–36 hours, 40 per cent at 37–48 hours and 12 per cent at 49–60 hours in a different study.^12^ It is common practice to inseminate animals 12 hours after observed oestrus, using the AM:PM rule; this was shown to be the optimum time for insemination to achieve both acceptable fertilisation rates and desirable embryo quality.^13^ The seven-day CIDR synchronisation protocol is still commonly used in the UK and is often combined with two inseminations on consecutive days. This is associated with increased cost but is also anecdotally considered to be associated with increased number of pregnancies per treatment (P/T). To the best of our knowledge this has yet to be proven. The objective of this study was to determine whether two inseminations (at 48 and 72 hours after CIDR withdrawal) will increase the P/T by optimising the timing of insemination of heifers coming into oestrus at different times after a seven-day CIDR (Eazi-Breed CIDR Cattle, Zoetis) protocol with PGF2α given on D6 postprogesterone implantation in dairy heifers in commercial UK dairy farms compared with inseminating once after the same protocol (at 56 hours).

## Materials and methods

The study was approved by the University of Liverpool Veterinary Research Ethics Committee (Reference Number: VREC286). Sample size calculations suggested that 169 animals per group would allow the detection of a 15 per cent difference in P/T (65 per cent v 50 per cent) using an α of 0.05 per cent and 80 per cent power. However, due to financial and time constraints, the authors were only able to enrol a total of 267 nulliparous, Holstein or Holstein-Friesian heifers from five herds in the South West of England, with a range of age of 388–736 days. The number of enrolled animals would allow the detection of a 17.5 per cent difference in P/T using an α of 0.05 per cent and 80 per cent power. All enrolled heifers were not inseminated previously. The study was conducted between November 2014 and February 2016. Heifers were not enrolled if they were discovered to be freemartins on D0. The diet for the previous eight-week period was recorded for each farm. Heifers enrolled in the study were kept under the same management conditions and fed on the same diet throughout the course of the study period (until date of pregnancy diagnosis) on a farm-by-farm basis.

A list of heifers from each farm was separately entered into Excel (Microsoft Office) and assigned random numbers (RAND function); the random numbers were then sorted into ascending order and the first half were assigned to group A and the second half to group B. This was done by a member of the research team.

All enrolled heifers received a progesterone intravaginal insert containing 1.38 g of progesterone (Eazi-Breed CIDR Cattle, Zoetis) on D0 (the CIDR plastic wire was trimmed by half to avoid herd mates pulling out the CIDR), and an intra-muscular injection of 0.15 mg d-cloprostenol (2 ml Prellim, Zoetis) on D6; the CIDR was withdrawn on D7 by the farmer. Heifers were then inseminated according to their group with time of CIDR withdrawal and time of insemination recorded for each group:

- Group A animals were inseminated twice, on the mornings of D9 and D10 as near to 48 and 72 hours after CIDR withdrawal as possible.
- Group B animals were inseminated once, on the afternoon of D9 as near to 56 hours after CIDR withdrawal as possible.

On D0 withers height (cm), heart girth (cm) and body condition score^14^ were recorded by the same researcher who was blinded to the treatment group each animal was assigned to. Height was measured with a vertical standard that was equipped with a crossbar with level.^15^ Heart girth measurements were used to estimate true weight (kg) using the method described by Heinrichs and Hargrove.^16^ The same researcher was scanning both left and right ovaries using ultrasound (BCF Technology linear probe, detailed mode, 8.5 MHz), and ovaries were mapped recording both largest follicle and corpus luteum (CL) diameter (measured in mm using acoustic enhancement). On study D6 the ovaries were scanned again recording the same measurements as above.

Heifers were inseminated using AI by either a trained herdsman or a technician. On each herd, only one person carried out the inseminations and this person was different between herds. The bull and breed were recorded with the same bull used for both groups A and B for one cohort of animals on the same herd; different bulls were used between different cohorts on the same herd and between herds. Sex sorted semen was used on one herd and conventional semen used on the remainder of herds.

Heifers were scanned at 38 days±3 days after study D10 using a BCF Easi-Scan with linear 4.5–8.5 Hz probe on ovary/early pregnancy mode to obtain the number of pregnancies per group. Pregnancy was confirmed by the presence of a viable embryo with a visible heartbeat that was surrounded by a clear black anechoic fluid. The person performing the scanning was blinded to the treatment groups. Heifers returning to oestrus before D38 were not reinseminated until after a negative pregnancy was confirmed on D38 to avoid the risk of damaging a viable conceptus that could potentially alter the final results.

### Statistics

All analyses were conducted using Minitab V.17. Differences between groups at enrolment were assessed using a two-sample *t* test for age, body condition score (BCS), withers height, weight, CL size on D0 and dominant follicle size on D0.

A binomial distribution was assumed for P/T, and a multivariable binary logistic regression model was used to assess the effect of herd, treatment group, age, withers height, weight, CL size on D0 and D6, dominant follicle size on D0 and D6, the presence of a CL on D0 and D6, and BCS on P/S. The final model was chosen by backward elimination, excluding any variables with a P value of greater than 0.1. Treatment group and herd were kept in the final model regardless of their P value.

Continuous variables of age, withers height and dominant follicle size on D6 were also categorised into quartiles. The continuous variables were then replaced by these categorical variables in the final multivariable binary logistic regression model, and assessments of odds ratios were made by category. Statistical significance was defined as P≤0.05 and statistical tendencies as 0.05<P≤0.10.

## Results

In total 237 heifers were included in the final analysis out of the 267 heifers that were originally enrolled. There were 119 heifers eligible from group A and 118 heifers from group B. Twenty-four animals were excluded from herd 1 because they were much older than other cohorts (mean age of 864 days v 467 days), they were inseminated using sexed semen, and the second cohort was mistakenly inseminated using a different bull’s semen for group B. Two out of the 24 had also lost their progesterone implants. Two heifers were excluded from herd 2 because they came into oestrus before FTAI and were inseminated early. Four heifers were excluded from herd 5 because they were inseminated with different semen.

Descriptive statistics and a two-sample *t* test revealed no significant difference between treatment groups at enrolment in terms of age, BCS, withers height, weight, CL size or dominant follicle size (table 1). The proportion of P/T group (±se) was 57.4±3.2 per cent for both groups: 54.6±4.6 per cent for group A (inseminated twice) and 60.2±4.5 per cent for group B (inseminated once).

**TABLE 1:** Mean (M), sd and range values of age, BCS, height, weight, corpus luteum (CL) size and dominant follicle size at enrolment for different treatment groups

Variable	Group	N	M	sd	Range	P value
Age (days)	A	119	468.7	39.1	398–617	0.586
	B	118	465.6	47.1	388–736	
BCS (0–5)	A	119	2.88	0.31	2.25–4.0	0.573
	B	118	2.90	0.30	2.0–4.0	
Height (cm)	A	119	127.9	4.8	115–138	0.110
	B	118	126.8	5.1	116–139	
Weight (kg)	A	119	408.0	58.6	314–586	0.169
	B	118	397.3	60.7	244–600	
CL size day 0 (mm)	A	95	21.8	5.5	10–38	0.531
	B	96	21.3	5.5	8–32	
Follicle size day 0 (mm)	A	107	12.16	3.20	8–22	0.762
	B	95	12.03	3.02	8–22	

A two-sample *t* test was used in order to assess the potential significance of any difference.

BCS, body condition score.

Variables retained in the final model included age, withers height, dominant follicle size on D6 and CL presence on D0. Multivariable logistic regression analysis revealed that herd and treatment group did not have a significant effect on P/T (P=0.284, P=0.510, respectively). Age (P=0.044) and dominant follicle size on D6 (P<0.001) were statistically significantly associated with P/T. The presence of a CL on D0 (P=0.082) and withers height (P=0.053) were not statistically significantly associated with P/T; a tendency towards significance was observed. More specifically, increased age at enrolment (odds ratio per unit change: 0.9925; 95 per cent confidence interval (CI): 0.9855 to 0.9997) and increased dominant follicle size on D6 (odds ratio per unit change: 0.8374; 95 per cent CI: 0.7614 to 0.9210) were associated with decreased P/T. Increased withers height (odds ratio per unit change: 1.096; 95 per cent CI: 0.9974 to 1.2047) was associated with increased P/T. Heifers with a CL on D0 were 1.951 (95 per cent CI: 0.917 to 4.150) times more likely to become pregnant than heifers without a CL. Heifers with the largest dominant follicle sizes (fourth quartile, 16–22 mm) were 5.54 times (95 per cent CI: 2.43 to 12.63) less likely to be pregnant than those heifers with the smallest dominant follicles on D6 (first quartile, 8–10 mm).

Proportion of P/T for different quartiles of the variables age, withers height, and dominant follicle size on D6 and for presence or not of a CL on D0, and odds ratios obtained from multivariable logistic regression modelling are presented in table 2.

**TABLE 2:** Proportion of pregnancies per treatments (P/T) for different quartiles of the variables age, withers height, and dominant follicle size on day 6 and for presence or not of a corpus luteum on day 0

Variable	Category	Range	P/T	Odds ratio	P value
Age (days)	1	388–438	0.61	0.99 (0.99–1)	0.044
	2	439–464	0.60		
	3	465–492	0.53		
	4	493–736	0.55		
Withers height (cm)	1	115–123	0.50	1.10 (1–1.20)	0.053
	2	124–128	0.57		
	3	129–131	0.56		
	4	132–139	0.66		
Presence of a corpus luteum on day 0 (mm)	Yes		0.60	1.95 (0.92–4.15)	0.082
	No		0.44	Reference	
Dominant follicle size on day 6 (mm)	1	8–10	0.67	0.84 (0.76–0.92)	<0.001
	2	11–12	0.64		
	3	13–15	0.60		
	4	16–22	0.31		

Odds ratios obtained from multivariable logistic regression modelling are also presented (odds of being pregnant v non-pregnant). Odds ratios for the continuous variables age, withers height and dominant follicle size on day 6 are presented per unit increase.

## Discussion

The current study did not show statistically significant differences between treatment groups A and B in terms of P/T. Inseminating animals twice added the extra cost of a second straw of semen, increased the number of labour hours required to separate out and inseminate the heifers and added one extra animal handling (which could also be associated with additional stress for the animal). Therefore, the results of this experiment do not support the need for a second insemination after a seven-day CIDR synchronisation protocol, a practice still commonly adopted by UK veterinarians and farmers. The farms used in this study would be considered typical UK dairy farms, and therefore our results could be generalised at least for the UK dairy heifers population.

Increased dominant follicle size on D6 had a detrimental effect on P/T regardless of the treatment group or herd, and this supports the results reported by Savio and others,^17^ who found persistence of the first dominant follicle reduced the pregnancy rate from 64.8 to 37.1 per cent in dairy heifers. Increased dominant follicle size is related to the duration of dominance of that ovulatory follicle, and Cerri and others^6^ showed that a reduction in the period of follicular dominance improved embryo quality. Austin and others^18^ reported that the optimal duration of dominance of a particular follicle should be ≤8 days, and fertility in heifers was greatest when duration of dominance was ≤5 days. The size of the dominant follicle is dependent upon the stage of the oestrous cycle when a synchronisation programme is started (Stevenson^12^). In the present study heifers were synchronised at random stages of the oestrous cycle, and this may account for the difference in P/T seen in heifers with dominant follicles greater than 15 mm in diameter and shows the importance of follicle turnover for improved pregnancy results when using CIDR synchronisation protocols.

The presence of a CL on D0 was associated with a substantial numeric increase in P/T (P/T was 60.4 per cent for the heifers with a CL on D0 and 44.4 per cent for the ones without a CL on D0). This difference was not statistically significant; a tendency towards significance was observed. This increase is in agreement with other studies that found that the presence of a CL during treatment with progestins was beneficial for pregnancy outcome.^19–21^ Although not measured in the current study, higher levels of progesterone in heifers with both an active CL and CIDR device are a possible explanation for this increase in fertility. van Werven and others^22^ showed that higher milk progesterone concentrations on the day of device insertion in cows predicted the P/AI outcome, with P/AI increasing linearly as P4 concentrations increased. The absence of a functional CL would lead to reduced circulating levels of progesterone in the presence of CIDR compared with those animals with a functioning CL^17^; reduced levels of progesterone decrease the negative feedback effect of progesterone on the hypothalamus, leading to increased luteinising hormone pulse frequencies (one pulse per 1–2 hours),^17 23^ maintaining the growth and oestradiol production of the dominant follicle.^7^ This leads to an extension in its period of follicullar dominance (persistence) and premature resumption of meiosis of the oocyte,^7 24^ all having detrimental effects on fertility. Admittedly, some of the heifers without a CL on D0 may also have been prepubertal at the start of the synchronisation protocol, and this would explain the reduction in P/T seen in this group compared with those with a CL. Reproductive tract scoring would have allowed a better assessment of pubertal status; unfortunately this assessment was not included in this study.^25^

Increased age in this study had a negative impact on P/T. This is in agreement with a large US study in which heifers receiving their first insemination between 15 and 16 months (450–480 days) of age had higher conception rates compared with those inseminated at more than 16 months (>480 days) of age and those more than 26 months (780 days) of age.^26^ The reason for this difference was not demonstrated in this study, but the researchers postulated this may have been caused by overconditioning in these older animals. Adamiak and others^27^ found that moderately conditioned heifers (BCS >3.5/5) fed a twice maintenance diet produced fewer oocytes and had lower blastocyst yield following in vitro fertilisation and embryo culture, which was associated with a hyperinsulinaemic state. Older heifers in the current study were indeed more likely to be overconditioned (31.5 per cent of the heifers in the highest age quartile had a BCS of 3.25 or higher; only 1.7 per cent of the heifers in the lowest age quartile had a BCS 3.25 or higher), and this may explain their lower fertility. Brickell and others^28^ showed calves that had larger heart girth measurements at 180 days and were heavier at 450 days required on average more services per conception, but found no correlation with insulin levels and fertility. The mean weight gain of heifers that required one insemination per conception was 0.81±0.03 kg/day compared with 0.91±0.05 kg/day for heifers that required more than two inseminations to conceive.

Increased withers height increased the chances of pregnancy. Brickell and others^28^ showed that larger heifers in terms of skeletal size measured by withers height (at 180 and 450 days) were on average younger at first breeding and at calving. Weight was not correlated with P/T in this study, in agreement with Chebel and others,^29^ who found no correlation between weight at the initiation of a breeding programme and conception rate at 40 days after AI in Holstein heifers.

In conclusion, inseminating heifers twice after a seven-day CIDR protocol achieved P/T results comparable to inseminating heifers once at 56 hours. Inseminating animals once reduced the number of animal handlings, semen costs and labour time required to carry out the synchronisation protocol while achieving acceptable P/T.
